# Ubiquitin-Like Protein SAMP1 and JAMM/MPN+ Metalloprotease HvJAMM1 Constitute a System for Reversible Regulation of Metabolic Enzyme Activity in Archaea

**DOI:** 10.1371/journal.pone.0128399

**Published:** 2015-05-26

**Authors:** Shiyun Cao, Nathaniel Hepowit, Julie A. Maupin-Furlow

**Affiliations:** 1 Department of Microbiology and Cell Science, University of Florida, Gainesville, Florida, United States of America; 2 Genetics Institute, University of Florida, Gainesville, Florida, United States of America; National Institute for Medical Research, Medical Research Council, London, UNITED KINGDOM

## Abstract

Ubiquitin/ubiquitin-like (Ub/Ubl) proteins are involved in diverse cellular processes by their covalent linkage to protein substrates. Here, we provide evidence for a post-translational modification system that regulates enzyme activity which is composed of an archaeal Ubl protein (SAMP1) and a JAMM/MPN+ metalloprotease (HvJAMM1). Molybdopterin (MPT) synthase activity was found to be inhibited by covalent linkage of SAMP1 to the large subunit (MoaE) of MPT synthase. HvJAMM1 was shown to cleave the covalently linked inactive form of SAMP1-MoaE to the free functional individual SAMP1 and MoaE subunits of MPT synthase, suggesting reactivation of MPT synthase by this metalloprotease. Overall, this study provides new insight into the broad idea that Ub/Ubl modification is a post-translational process that can directly and reversibly regulate the activity of metabolic enzymes. In particular, we show that Ub/Ubl linkages on the active site residues of an enzyme (MPT synthase) can inhibit its catalytic activity and that the enzyme can be reactivated through cleavage by a JAMM/MPN+ metalloprotease.

## Introduction

The post-translational covalent attachment of ubiquitin/ubiquitin-like (Ub/Ubl) proteins to protein targets has important roles in diverse cellular activities, such as proteasome-dependent protein degradation, DNA repair, protein trafficking and autophagy [1–3]. Misregulation of the Ub/Ubl systems is implicated in a number of human diseases and, thus, is intensely studied. Recent knowledge regarding Ub/Ubl systems is importantly enhanced by discovery of Ubl modifier proteins in bacteria [4] and archaea [5], thus, increasing awareness of the diversity of this system to all three domains of life.

In archaea, Ubl proteins called SAMPs (small archaeal modifier proteins) were discovered in 2010 [5]. Like the Ub system, the C-terminal glycine of the SAMPs is conjugated to the lysine residues of numerous protein substrates by a mechanism requiring the E1-like enzyme, UbaA [5–7]. SAMP modification (sampylation) can be cleaved and removed by a JAMM/MPN+ domain metalloprotease, HvJAMM1 [7–9].

Recent studies of *Haloferax volcanii* reveal 45 lysine residues are targets of sampylation, as mapped by tandem mass spectrometry (LC-MS/MS) analysis of SAMP-conjugates [5, 7, 8][Wu, unpublished). Interestingly, many of these target lysine residues are in close proximity to putative active site residues of enzymes [*e*.*g*., ribose-1,5-bisphosphate isomerase, molybdopterin (MPT) synthase large subunit (MoaE), isochorismatase family cysteine hydrolase and an OsmC-like peroxidase]. These findings suggest enzyme activity could be inhibited by sampylation. Considering the desampylation activity of HvJAMM1, we were motivated to investigate whether the archaeal Ubl SAMPs and a JAMM/MPN+ protease (like HvJAMM1) really constitute a regulating system for enzyme inhibition and activation. In this research, we used MPT synthase as model system to explore this question.

Molybdenum cofactor (MoCo) is essential to form the catalytic center of diverse enzymes, and MoCo biosynthesis is a conserved pathway that exists in all domains of life [10]. MPT synthase catalyzes the biosynthesis of MPT, a precursor of MoCo. In bacteria, MoaD and MoaE are the small and large subunits of the heterotetrameric MPT synthase, respectively [11]. The C-terminal glycine residue of MoaD is adenylated by MoeB [12], a member of the E1/MoeB/ThiF superfamily. Adenylated MoaD is subsequently thiocarboxylated by relay of sulfur from cysteine desulfurase (IscS) and rhodanese domain (YnjE) [13, 14] proteins. The C-terminal glycine of thiocarboxylated MoaD (MoaD-COSH), which has the activated sulfur, is inserted into the active site of MoaE, thus, enabling the catalytic transfer of sulfur from MoaD to precursor Z to form MPT [11, 14]. Archaea encode homologs of the bacterial MoeB (UbaA), MoaD (SAMP1) and MoaE and, thus, are presumed to perform similar functions. Our previous study of *Hfx*. *volcanii*, showed UbaA, SAMP1 and MoaE are essential for MoCo-dependent DMSO reductase activity and are required for growth under conditions requiring MoCo [15], suggesting function in MoCo biosynthesis.

Here, we demonstrate that MPT synthase activity is inhibited by the covalent attachment of Ubl SAMP1 to MoaE. We also show the JAMM/MPN+ metalloprotease HvJAMM1 can cleave the covalently linked inactive SAMP1-MoaE to free intact functional SAMP1 and MoaE and reactivate MPT synthase. Thus, JAMM/MPN+ proteases can function with Ub/Ubl proteins as a regulating system for enzyme inhibition and activation and, therefore, correspondingly can regulate cellular activity.

## Results

### SAMP1-MoaE linear fusion is cleaved by HvJAMM1 *in vivo*

During our investigation of the distribution and genetic linkage of *ubl* and *moaE* homologs, we noticed that many bacteria, a number of archaea and a few eukaryotes encode Ubl-MoaE domain fusions (S1 Table) (in this context Ubl represents an N-terminal domain with a predicted β-grasp fold similar to Ub). Interestingly, archaeal Ubl-MoaE fusions appeared restricted to the phylum Crenarchaeota, while bacterial representatives were widespread including those of Deinococcus-Thermus, Chloroflexi, Fibrobacteres/Acidobacteria, Firmicutes, Proteobacteria and Actinobacteria [with the Ubl-MoaE fusion of *Mycobacterium tuberculosis* previously reported [16]]. These Ubl-MoaE fusions are predicted to be inactive, since the C-terminal glycine residue of the Ubl domain would not be accessible for thiocarboxylation and, thus, unable to complete the biochemical reaction of transferring sulfur to the precursor Z to form MPT. However, this prediction has yet to be tested. Our previous *in vitro* study with purified components showed that a linear fusion of the Ubl SAMP1 with MoaE (SAMP1-MoaE) can be cleaved by HvJAMM1 into free SAMP1 and MoaE, but activity and cleavage site of the protein products were not demonstrated [8].

To further investigate, whether or not this cleavage can occur *in vivo*, we expressed SAMP1-MoaE ectopically as a linear fusion in *Hfx*. *volcanii* strains with and without the *jamm1* gene encoding JAMM/MPN+ metalloprotease HvJAMM1. Protein fusions included SAMP1-MoaE as well as its protein variants SAMP1_ΔGG_-MoaE and SAMP1_ΔVSGG_-MoaE (where _ΔGG_ and _ΔVSGG_ are respective deletions of the C-terminal-GlyGly and-ValSerGlyGly residues of SAMP1). An empty vector was used as a negative control. In addition, the strains had a deletion of the E1-like *ubaA* gene to reduce the complexity of MoaE conjugates otherwise formed through sampylation. N-terminal Flag (Flag-) and C-terminal StrepII (-StrepII) tags were fused to SAMP1 and MoaE, respectively, to specifically detect the proteins of interest by immunoblotting. SAMP1 and MoaE are previously demonstrated to be functional with these tags based on our finding that the encoding genes complement their respective *Δsamp1* and *ΔmoaE* mutant strains for MoCo-dependent DMSO reductase activity and anaerobic growth with DMSO as an electron acceptor (the growth condition requiring MoCo biosynthesis) [15].

**Fig 1:**
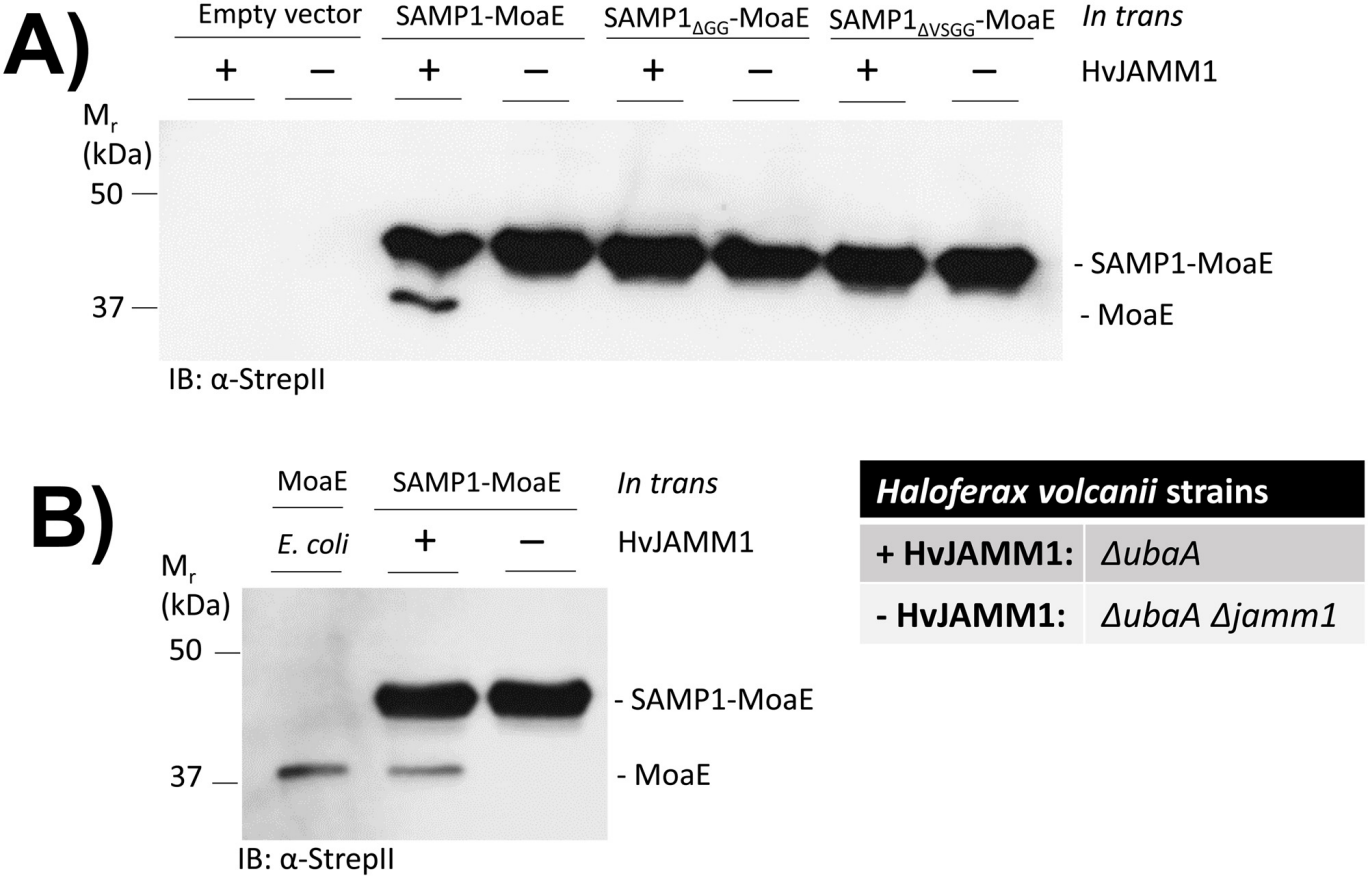
SAMP1-MoaE linear fusion is cleaved by HvJAMM1 *in vivo*. *In trans* expression of linear fusions SAMP1-MoaE, SAMP1_ΔGG_-MoaE (SAMP1 C-terminal-GlyGly deleted), and SAMP1_ΔVSGG_-MoaE (SAMP1 C-terminal-ValSerGlyGly deleted) in HvJAMM1 positive (*ΔubaA*) and negative (*ΔubaAΔjamm1*) strains. Empty vector **(A)** and *E*. *coli* produced MoaE-StrepII **(B)** were used as controls. SAMP1 and MoaE were fused at N- and C-terminal residues to Flag- and StrepII-tags, respectively. *Hfx*. *volcanii* strains were grown aerobically to stationary phase in ATCC974 medium. Cell lysates were separated by reducing SDS-PAGE and analyzed by α-StrepII immunoblotting.

With this approach, cleavage of the SAMP1-MoaE fusion was detected *in vivo* and found to be dependent on HvJAMM1. In particular, the cleavage product specific for the MoaE fragment of the SAMP1-MoaE fusion was detected in the HvJAMM1 positive (parent) strain but not in the HvJAMM1 (*Δjamm1*) mutant strain (Fig 1). By contrast, cleavage of the SAMP1_ΔGG_-MoaE and SAMP1_ΔVSGG_-MoaE fusions was not detected by this assay (Fig 1). Thus, we conclude that the SAMP1-MoaE linear fusion is cleaved by HvJAMM1 not only *in vitro* [8] but also *in vivo* (this study) and that the C-terminal residues of SAMP1 are important determinants for detecting this cleavage activity in the cell.

### HvJAMM1 cleavage of SAMP1-MoaE fusion generates products of the same molecular mass as intact functional SAMP1 and MoaE

**Fig 2:**
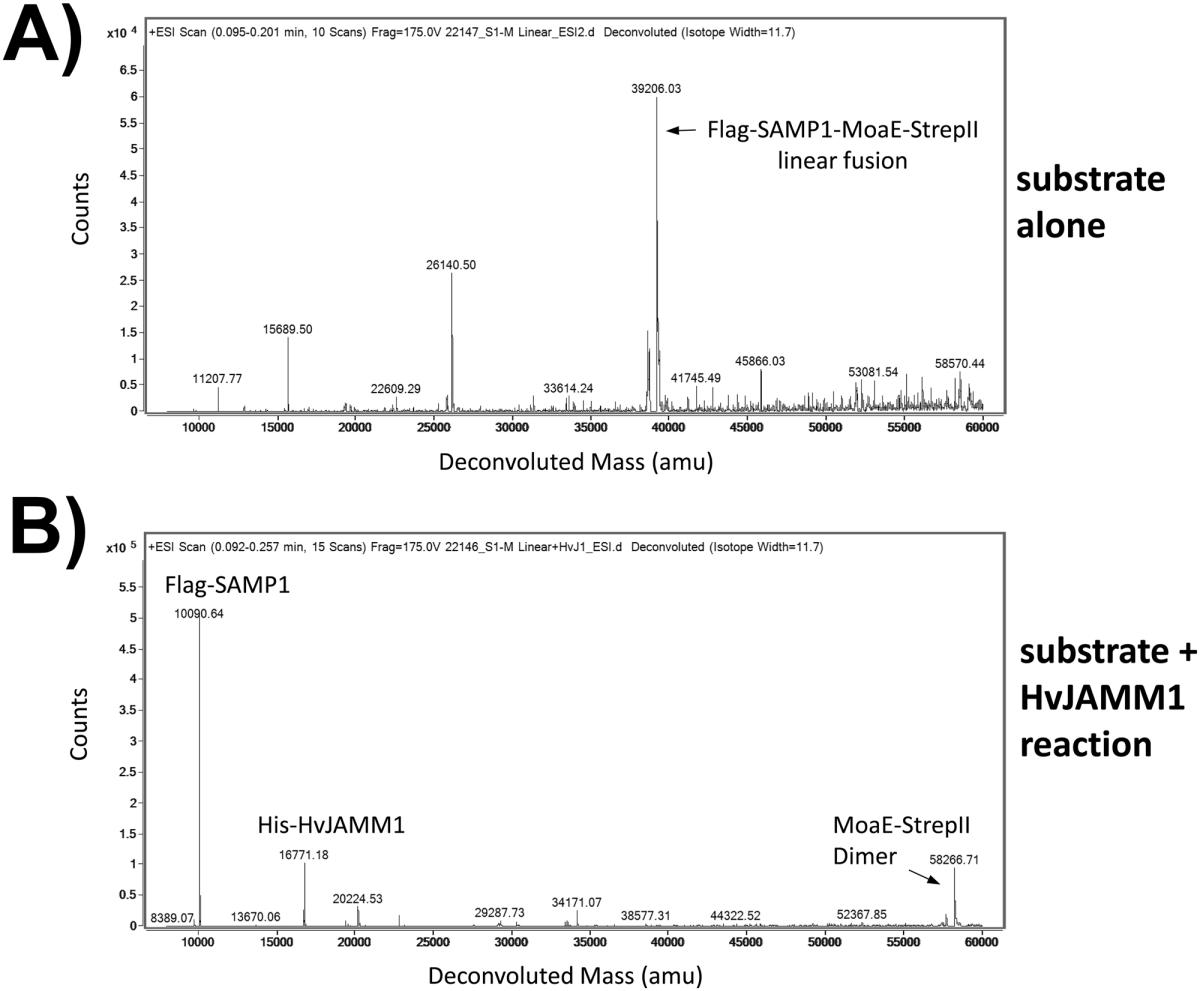
Deconvoluted spectra of SAMP-MoaE linear fusion cleaved by HvJAMM1. Substrate (Flag-SAMP1-MoaE-StrepII linear fusion protein, represented as SAMP1-MoaE) was incubated in the absence **(A)** and presence **(B)** of HvJAMM1 at 50°C for 11 h (see methods for details). Samples were dialyzed against water and analyzed by ESI-MS. Theoretical molecular masses calculated as 39,205.07 Da for Flag-SAMP1-MoaE-StrepII linear fusion, 10,089.9 Da for Flag-SAMP1, 16,771.17 Da for His-HvJAMM1, 29,133.1 Da and 58,266.2 Da for the monomeric and dimeric forms of MoaE-StrepII. amu, atomic mass unit.

To further investigate whether the SAMP1 and MoaE proteins that are released from a SAMP1-MoaE linear fusion by HvJAMM1 are same as the intact functional SAMP1 and MoaE, we performed an *in vitro* desampylation assay followed by electrospray ionization mass spectrometry (ESI-MS) analysis of the products. Reactions included purified protein substrate (Flag-SAMP1-MoaE-StrepII, referred to as SAMP1-MoaE linear fusion) incubated with and without enzyme (HvJAMM1). After dialysis into water, the samples were immediately analyzed by ESI-MS. For the substrate-only control, the accurate mass of the SAMP-MoaE linear fusion was detected as the main signal with no free SAMP1 or MoaE products observed (Fig 2A). The HvJAMM1-catalyzed reaction, by contrast, revealed the major ESI-MS signals corresponded to the accurate masses of HvJAMM1, SAMP1, and a dimer of MoaE (Fig 2B) (including affinity tags, where the accurate mass of HvJAMM1 alone was previously determined [8]). The MoaE dimer is biologically relevant, since MoaE in MPT synthase also exists as a dimer [11]. Based on these results, HvJAMM1 can cleave the SAMP1-MoaE fusion precisely after the C-terminal Gly of SAMP1 and generate SAMP1 and MoaE as free and intact polypeptides that could be functional.

### HvJAMM1 metalloprotease cleaves SAMP1-MoaE into active MPT synthase

To determine whether or not the HvJAMM1 metalloprotease cleaves a SAMP1-MoaE fusion into individual subunits that form an active MPT synthase complex, we used an *in vivo* approach. Detecting enzyme-mediated activation of a SAMP1-MoaE fusion is biologically relevant, as many organisms are predicted to encode a single polypeptide with a tandem arrangement of Ubl and MoaE domains based on genome sequence (S1 Table). In *Hfx*. *volcanii*, the MPT synthase homologs (SAMP1 and MoaE) are needed for DMSO respiration and reductase activity [15], presumably because the MoCo active site of the terminal DMSO reductase requires formation of MPT. Anaerobic growth with DMSO as a terminal electron acceptor can, therefore, be used as an assay to monitor the activation of MPT synthase by HvJAMM1. In our *in vivo* assay, the C-terminal glycine of SAMP1 was covalently linked through a linear peptide bond to the N-terminus of MoaE and, thus, was predicted to be inaccessible for thiocarboxylation and sulfur relay to form MPT without proteolytic cleavage and exposure of the C-terminal glycine. In *Hfx*. *volcanii*, *samp1* and *moaE* are expressed as separate genes; therefore, the SAMP1-MoaE linear fusion was ectopically expressed in a *Δsamp1ΔmoaE* mutant background.

**Fig 3:**
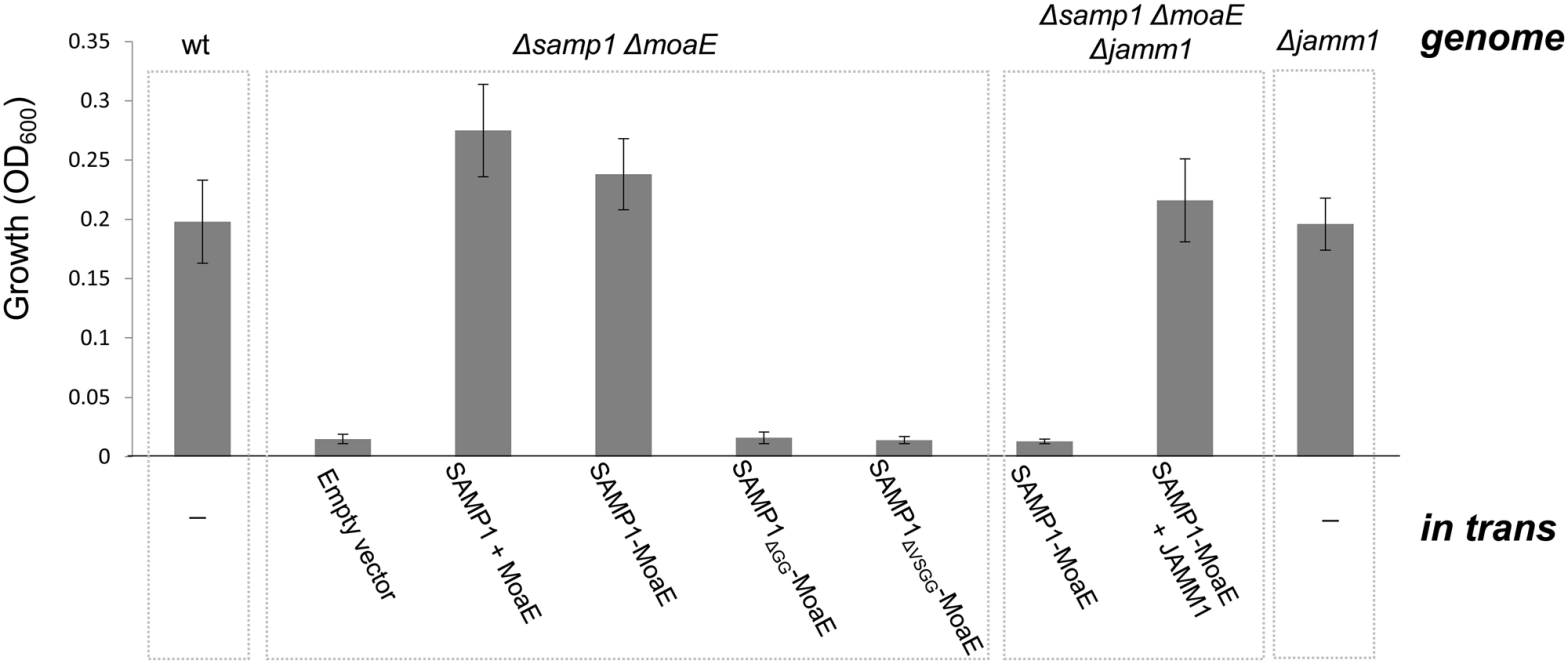
HvJAMM1 is required for SAMP1-MoaE to complement a *Δsamp1ΔmoaE* mutant strain for DMSO respiration. Strains were grown anaerobically in YPC medium with DMSO as electron acceptor. Growth was monitored by cell density (OD_600_ units at 70 h). SAMP1 + MoaE and +JAMM1 represent separately expressed proteins, while SAMP1-MoaE and its variants are linear protein fusions. ΔGG and ΔVSGG are C-terminal deletions of SAMP1.

HvJAMM1 was found to cleave SAMP1-MoaE into an active MPT complex based on the following *in vivo* evidence. Importantly, expression of the SAMP1-MoaE linear fusion *trans*-complemented the *Δsamp1ΔmoaE* mutant for DMSO respiration, but only when the strain carried a *jamm1*+ allele not a *Δjamm1* deletion (Fig 3). The *Δsamp1ΔmoaEΔjamm1* triple mutant could be complemented for DMSO respiration by expression of the SAMP1-MoaE linear fusion with re-integration of the *jamm1+* gene onto the *Hfx*. *volcanii* genome (Fig 3). Inclusion of coding sequence for a C-terminal StrepII tag on *jamm1* had no impact on this complementation (Fig 3). For controls, SAMP1 and MoaE were co-expressed as separate genes and found to complement the *Δsamp1ΔmoaE* mutant for DMSO respiration, while *Δsamp1ΔmoaE* mutant strains carrying an empty vector displayed no growth under similar growth condition (Fig 3). Thus, we demonstrate that the SAMP1-MoaE linear fusion is inactive in DMSO respiration and requires the metalloprotease HvJAMM1 to complement a *Δsamp1ΔmoaE* mutant for this MPT-dependent process. HvJAMM1 mediated cleavage of SAMP1-MoaE is likely correlated with the observed complementation and associated with the formation of an active MPT synthase complex.

### Lysine 240 is a crucial residue of the MoaE active site

**Fig 4:**
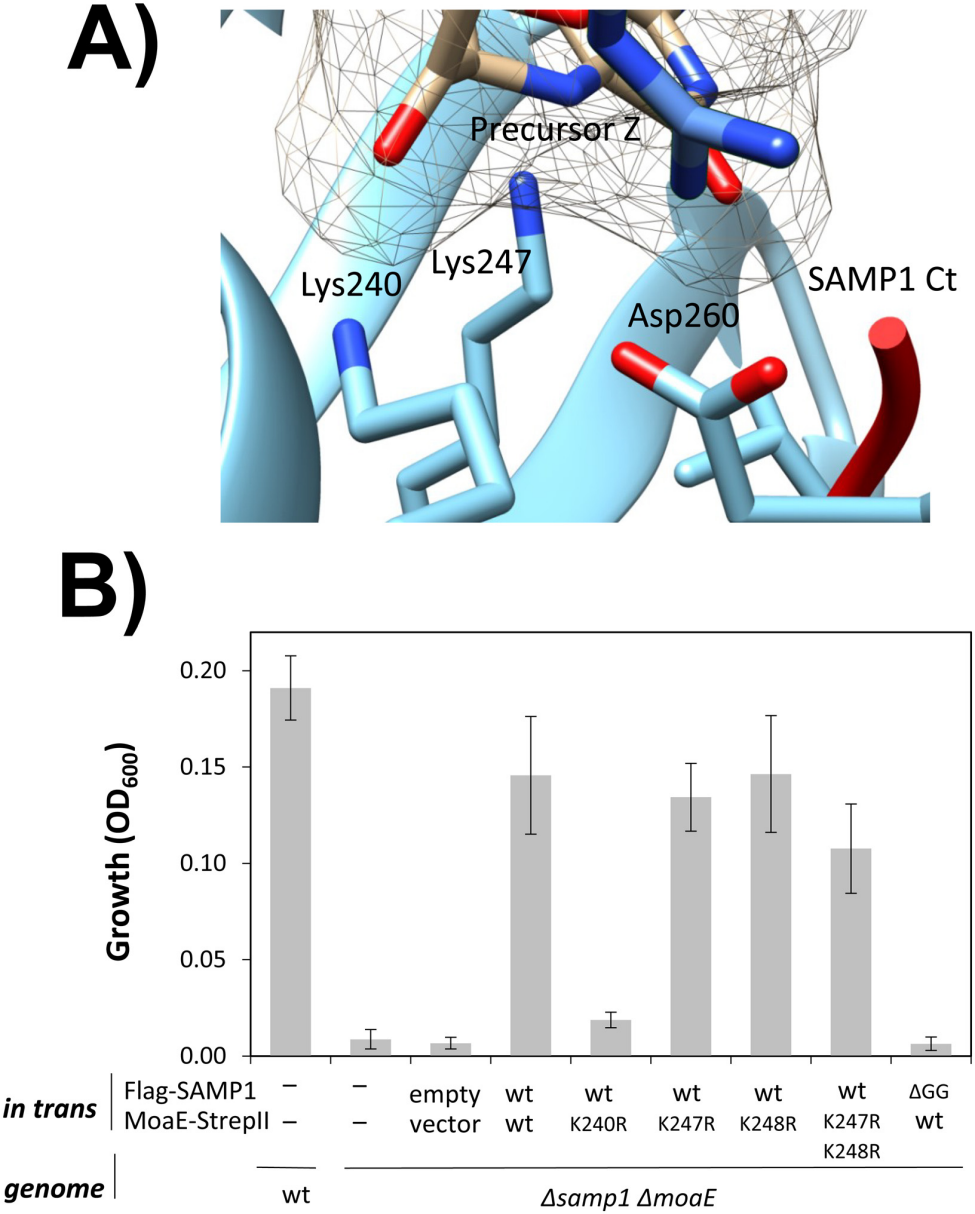
Lys_240_ is the active site of MoaE. **(A)** Model of the active site of MPT synthase in complex with its substrate precursor Z. The MPT synthase large subunit (MoaE, blue) and C-terminal tail of the small subunit (SAMP1 Ct, red) are represented as ribbon diagrams. **(B)** Strains were grown anaerobically in YPC medium with DMSO as electron acceptor. Growth was monitored by cell density (OD_600_ units at 70 h).

**Fig 5:**
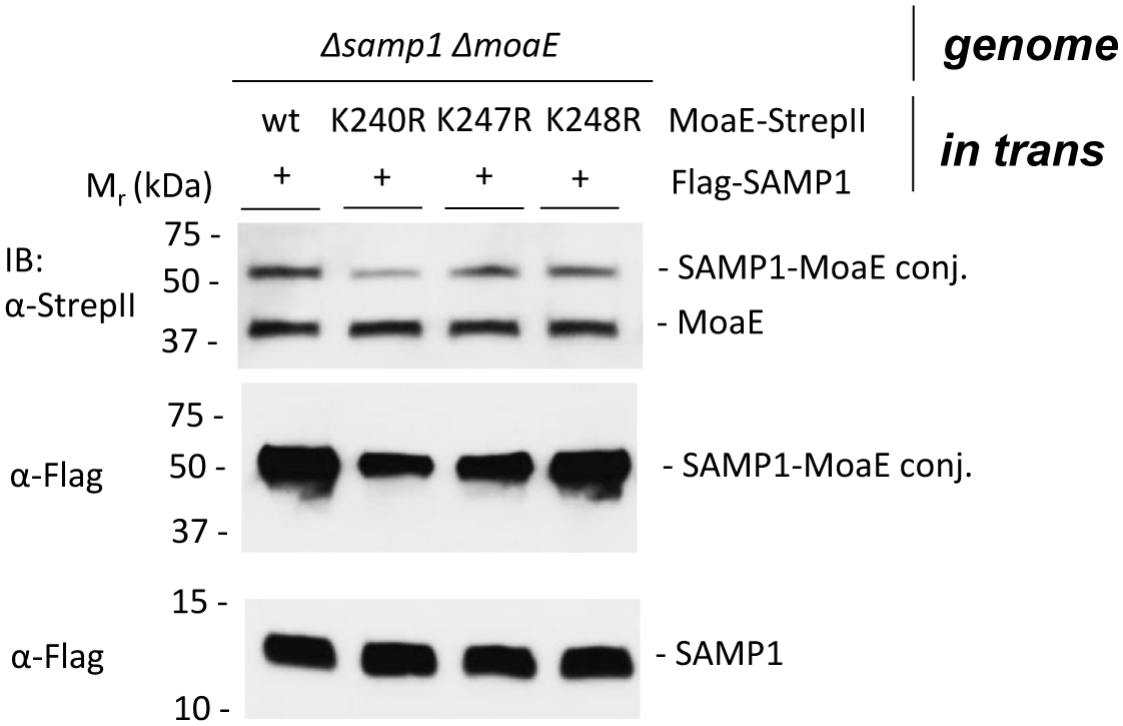
MoaE_K240R_ reduces but does not abolish formation of SAMP1-MoaE conjugates. *In trans* expression of tandem Flag-SAMP1 (wt as a negative control) and MoaE-StrepII (wt and different Lys mutated variants) in *Δsamp1ΔmoaE* mutant strain. Strains were grown aerobically to stationary phase in ATCC974 medium. Cell lysates were separated by SDS-PAGE and analyzed by α-Flag and α-StrepII immunoblotting.

*Hfx*. *volcanii* MoaE has a number of lysine residues (K139, 160, 175, 183, 240, 247 and 248), and, based on homology modeling, K240 and K247 are analogous to the active site residues of bacterial MoaE [8]. To test whether K240 and K247 are really active site residues of the *Hfx*. *volcanii* MoaE, we performed site direct mutagenesis (SDM) by conservative replacement of the lysine residues with arginine. We then examined whether or not expression of the MoaE variants would complement a *ΔmoaE* mutation for DMSO respiration. The results showed that, while MoaE_K240R_ was expressed, this variant lost the ability to complement the *ΔmoaE* mutant for growth under conditions with DMSO as the terminal electron acceptor (Figs 4 and 5). By contrast, MoaE K247R and K248R were comparable to wild type in their ability to complement the *ΔmoaE* mutant for anaerobic growth (Fig 4). Thus, K240 appears to be a crucial residue of the active site of archaeal MoaE. We suggest that K240 harbors the side chain directly responsible for transferring sulfur from the C-terminal thiocarboxyl group of SAMP1 to precursor Z based on structural analogy to K119 of bacterial MoaE that is found essential for MPT synthase activity [17]. While K247 does not appear important for activity of the archaeal MoaE, the structurally analogous K126 of bacterial MoaE is found crucial for MPT synthesis with catalytic activity of the K126A variant nearly 60-times slower than wild-type [17]. We cannot rule out that differences in method are accountable for this contrast in measurable activity with the archaeal MoaE K247R variant monitored *in vivo* and the bacterial MoaE K126A variant monitored *in vitro*. However, enzyme evolution may also be responsible for these dissimilarities. *Hfx*. *volcanii* and *Escherichia coli* MoaE homologs display only 38% amino acid identity. Furthermore, *Hfx*. *volcanii* MoaE is a tandem MobB-MoaE domain fusion, while *E*. *coli* MoaE is a single domain protein.

### MoaE_K240_ is isopeptide linked to SAMP1 based on *in vivo* assay

Based on previous LC-MS/MS analysis, MoaE K240 and K247 residues are found isopeptide linked to the C-terminal glycine residues of the Ubl-proteins SAMP1/3 [7, 8]. To further examine these findings by a more direct *in vivo* assay, Lys to Arg variants of MoaE were generated and tested for their ability to form SAMP1 conjugates. MoaE variants included K240R, K247R and K248R, with the latter residue modeled in close proximity to the active site. If SAMP1 was covalently bound to MoaE at the lysine residues identified by MS/MS analysis, then these site-directed alterations were predicted to reduce the level of SAMP1-MoaE conjugates formed in the cell. Immunoblotting analysis showed that the level of SAMP1 conjugates was reduced for the MoaE K240R variant compared to wild type (Fig 5), consistent with K240 as a site of samp1ylation. SAMP1 conjugates of the K247R and K248R variants were at reduced levels compared to wild type but this reduction was not as pronounced as for K240R. The availability of other Lys residues on the MoaE variants for linkage to SAMP1 is the most likely explanation for why the level of conjugates of the MoaE Lys to Arg variants was not abolished. However, our results do provide further evidence that SAMP1 is covalently linked by an Ubl-isopeptide bond to the active site residue of MoaE (K240). Our previous *in vitro* study already showed that HvJAMM1 could cleave the SAMP1-MoaE conjugates to free SAMP1 and MoaE [8], suggesting desampylation activates MoaE.

## Discussion

In this study, an archaeal Ubl protein (SAMP1) and a JAMM/MPN+ metalloprotease (HvJAMM1) are shown to constitute a regulating system for the inhibition and activation of MPT synthase (SAMP1/MoaE complex). Our findings are relevant to the broad idea that Ub/Ubl proteins can inhibit an enzyme by covalent modification of active site Lys residues with subsequent proteolytic cleavage and reactivation by a JAMM/MPN+ metalloprotease such as HvJAMM1. This type of reversible post-translational modification system provides the organism with an advantage to rapidly regulate enzyme activity based on its demand and could be genetically modified to serve as a switch that directs the carbon flow of microbial biocatalysts in biotechnology applications. The sampylation system that controls MPT synthase in haloarchaea includes an apparent second level of regulation which is the covalent attachment of SAMP3 to MoaE [7]. Unlike SAMP1, SAMP3 is not essential for DMSO reductase activity, and the encoding gene does not complement a *Δsamp1* mutant for DMSO respiration [15]. Thus, SAMP3 is not directly involved in MPT biosynthesis. However, similarly to SAMP1, SAMP3 is covalently bound to the active site residue of MoaE (K240) when the demand for MPT is low (*i*.*e*., aerobically) [7], suggesting inhibition of MPT synthase by two distinct Ubl proteins. HvJAMM1 can cleave SAMP3- and SAMP1-MoaE conjugates [7, 8], suggesting enzyme reactivation after both types of post-translational modifications.

Eukaryotes also appear to use Ubl protein modifications for reversible regulation of catalytic enzyme activity. Recently, HMGS-1 (the 3-hydroxy-3-methyl-glutaryl-CoA synthase in the mevalonate pathway of *Caenorhabditis elegans*) was reported to be regulated by the Ubl SUMO and its corresponding cysteine-type protease ULP-4 [18]. HMGS-1 is sumoylated *in vivo*, which is thought to inactivate this enzyme, although the mechanism of how sumoylation can achieve this inactivation at the molecular level is still unclear. The level of HMGS-1 sumoylation is found to obviously increase in an *ulp-4* protease mutant and a partial rescue of the *ulp-4* mutant phenotype by mevalonate supplementation suggests HMGS-1 is less active when oversumoylated, placing the ULP-4 protease as an activator. This finding provides another example to support the model that regulation of metabolic enzymes can be performed by Ubl protein modification and removal.

## Materials and Methods

### Strains and growth conditions

Strains and primers used in this study are summarized in S2 and S3 Tables. *E*. *coli* strains were grown aerobically in Luria-Bertani medium at 37°C. *Hfx*. *volcanii* strains were grown aerobically in 42°C either in ATCC974 or YPC medium. For anaerobic growth with DMSO, strains were grown aerobically to log-phase (in 4 ml medium in 13 x 100 mm culture tube) and inoculated at 1% (vol/vol) for anaerobic growth with YPC medium plus 100 mM DMSO as a terminal electron acceptor (filled up to the brim in 15 x 100 mm screw-capped tubes). Ampicillin (0.1 mg∙ml^-1^), kanamycin (50 μg∙ml^-1^) and novobiocin (0.1 μg∙ml^-1^) were added as needed. Cells were grown aerobically in liquid medium with rotary shaking (200 rpm) or on plates (solid medium with 1.5% agar).

### Generation of mutant strains and site-directed mutagenesis

Mutant strains were generated by a *pyrE2*-based deletion method [19] and were identified by polymerase chain reaction (PCR) using primer pairs specific to the genomic region of the target gene and not the plasmid DNA used for generating the deletion. Amino acid exchange was performed by inverse PCR-based mutagenesis using Phusion DNA polymerase. DpnI-treated PCR amplicons were phosphorylated by T4 polynucleotide kinase and circularized by T4 DNA ligase prior to transformation into *E*. *coli* TOP10. DNA sequence of *trans*-expressed genes and PCR products derived from mutant strains was confirmed by Sanger automated DNA sequencing using an Applied Biosystems model 3130 genetic analyzer (ICBR Genomics Division, University of Florida).

### Mass spectrometry analysis of HvJAMM1-mediated desampylation

For SAMP1-MoaE substrate, the Flag-SAMP1-MoaE-StrepII linear fusion was expressed in *E*. *coli* Rosetta(DE3) strain using a pET24b-based plasmid by IPTG induction similarly to previously described [8]. Substrate protein was purified by tandem (StrepTactin and anti-Flag) affinity chromatography as previously described [8] with the addition of third step of purification by StrepTactin chromatography. Purified substrate was dialyzed into assay buffer (50 mM HEPES, pH 7.5, 2 M NaCl) and concentrated to 80 μM by use of Amicon Ultra-10 centrifugal filters (EMD Millipore, Darmstadt, Germany). For enzyme, the N-terminal His-tagged HvJAMM1 was expressed in *E*. *coli* Rosetta(DE3) strain with IPTG induction and purified by His-trap chromatography followed by gel filtration as previously described [8]. HvJAMM1 was concentrated to 100 μM by use of Amicon Ultra-3 centrifugal filters. For desampylation assay, 10 μl HvJAMM1 and 45 μl substrate were mixed together in a 1:3.6 molar ratio and incubated at 50°C for 11 h. For control, substrate alone (15 μl) was incubated under the same assay condition. Samples were dialyzed into water overnight by using mini dialysis tube (1 kDa MW cut-off, GE Healthcare) and analyzed by ESI mass spectrometry on the same day. Samples were treated with acetonitrile (50% v/v) and formic acid (1% v/v) and immediately loaded into Agilent 6210 time-of-flight mass spectrometer (Agilent Technologies, Inc., Santa Clara, California) with electrospray ionization source in positive mode for the determination of accurate mass.

### Immunoblotting

Cell pellets were boiled in SDS-reducing buffer for 20 min and centrifuged at 14,000 x *g* for 10 min at RT to obtain the supernatant. Samples were separated by 12% SDS-PAGE and transferred to PVDF membranes. Affinity tagged proteins were detected on membranes by immunoblotting with alkaline phosphatase-linked anti-Flag M2 monoclonal antibody (Sigma) and with mouse anti-StrepII polyclonal antibody (QIAGEN) followed by goat anti-mouse IgG (whole molecule)-alkaline phosphatase-linked antibody (Sigma). Alkaline phosphatase activity was detected by chemiluminescence using CDP-Star (Applied Biosystems) with X-ray film (Hyperfilm; Amersham Biosciences).

## Supporting Information

S1 Table Ubl-MoaE linear fusion proteins and JAB1/MPN+/MOV34 (JAMM) proteins exist in diverse species in all domains of life. (PDF)

S2 Table Strains and plasmids used in this study. (PDF)

S3 Table Primers used in this study. (PDF)
