# Supplementary material for: Ubiquitin-Like Protein SAMP1 and JAMM/MPN+ Metalloprotease HvJAMM1 Constitute a System for Reversible Regulation of Metabolic Enzyme Activity in Archaea
Source: PLoS One. 2015 May 26;10(5):e0128399. doi: 10.1371/journal.pone.0128399 (PMC4443979; doi:10.1371/journal.pone.0128399)
Supplement: S1 Table — (PDF) [file pone.0128399.s001.pdf]

S1 Table. Ubl-MoaE linear fusion proteins and JAB1/MPN+/MOV34 (JAMM) proteins exist in diverse species in all domains of life[a].

Species	Interpro Accession/ORF No.	
	**Ubl-MoaE linear fusion**	**JAB1/MPN+/MOV34 (JAMM)**
**Eukaryote**		
Amphimedon queenslandica (Sponge)	I1FSK0	6 ORFs
Capsaspora owczarzaki (strain ATCC 30864)	E9C7U2	5 ORFs
Strongylocentrotus purpuratus (Purple sea urchin)	W4Z164	7 ORFs
**Archaea (phylum Crenarchaeota)**		
Acidianus hospitalis (strain W1)	F4B937	F4B6Q4
Acidilobus saccharovorans (strain DSM 16705 / VKM B-2471 / 345-15)	D9PZU8	**D9Q1Q4**
Aeropyrum camini SY1 = JCM 12091	U3T8S8	U3TF61
Aeropyrum pernix (strain ATCC 700893 / DSM 11879 / JCM 9820 / NBRC 100138 / K1)	Q9YEH3	Q9YE90
Caldisphaera lagunensis (strain DSM 15908 / JCM 11604 / IC-154)	L0A8K6	L0AA61
Ignicoccus hospitalis	A8A942	-
Ignisphaera aggregans (strain DSM 17230 / JCM 13409 / AQ1.S1)	E0SRF3	E0SQK2
Metallosphaera cuprina (strain Ar-4)	F4FZ56	*
Metallosphaera sedula (strain ATCC 51363 / DSM 5348)	A4YEG8	A4YIK1
Metallosphaera yellowstonensis MK1	H2C334	H2C8V5 (80 aa – seems truncated at N-termius)
Pyrobaculum aerophilum (strain ATCC 51768 / IM2 / DSM 7523 / JCM 9630 / NBRC 100827)	Q8ZYL1	**Q8ZW14**
Pyrobaculum arsenaticum (strain DSM 13514 / JCM 11321)	A4WNE4	A4WML4
Pyrobaculum calidifontis (strain JCM 11548 / VA1)	A3MY34	A3MUW9
Pyrobaculum islandicum (strain DSM 4184 / JCM 9189)	A1RS95	**A1RVK1**
Pyrobaculum neutrophilum (strain DSM 2338 / JCM 9278 / V24Sta)	B1YC61	B1Y8Y1
Pyrobaculum oguniense (strain DSM 13380 / JCM 10595 / TE7)	H6QDQ2	H6Q636
Pyrobaculum sp. 1860	G7VBB8	G7VFA2
Pyrolobus fumarii (strain DSM 11204 / 1A)	**G0EF74**	**G0EF75**
Sulfolobales archaeon AZ1	W7KWC3	**W7L8T2**
Sulfolobus acidocaldarius Ron12/I	M1J1N3	M1JDD6
Sulfolobus islandicus (strain L.S.2.15 / Lassen #1)	C3MK74	C3MJ79
Sulfolobus solfataricus (strain 98/2)	D0KV26	D0KRG0
Sulfolobus tokodaii (strain DSM 16993 / JCM 10545 / NBRC 100140 / 7)	F9VN24	*
Thermoproteus tenax (strain ATCC 35583 / NBRC 100435 / JCM 9277 / Kra 1)	G4RP93	**G4RP39**
Thermoproteus uzoniensis (strain 768-20)	F2L436	**F2L2E7**

**Bacteria**		
Acidobacterium capsulatum (strain ATCC 51196 / DSM 11244 / JCM 7670)	C1F3F4	C1F3V2
Alicyclobacillus acidocaldarius (strain Tc-4-1)	F8IKK9	F8IDI2
Alicyclobacillus hesperidum URH17-3-68	J9HFF2	J9HBU7, J9HRP0
Anaerolinea thermophila (strain DSM 14523 / JCM 11388 / NBRC 100420 / UNI-1)	E8MY42	E8N4R5, **E8N0S3**
Anaeromyxobacter dehalogenans (strain 2CP-1 / ATCC BAA-258)	B8JGC1	B8J8K0, B8JFB0
Brevibacillus agri BAB-2500	L5MX53	L5MQ56
Brevibacillus borstelensis AK1	M8E9G6	-
Brevibacillus brevis (strain 47 / JCM 6285 / NBRC 100599)	C0ZHP4	C0ZAA2
Brevibacillus laterosporus LMG 15441	F7TRG4	H0U843
Conexibacter woesei (strain DSM 14684 / JCM 11494 / NBRC 100937 / ID131577)	D3F0J3	D3F2A1
Corallococcus coralloides (strain ATCC 25202 / DSM 2259 / NBRC 100086 / M2)	H8MZ90	H8MSK2
Cystobacter fuscus DSM 2262	S9P2I4	**S9QD14**
Deinococcus deserti (strain VCD115 / DSM 17065 / LMG 22923)	C1CYQ1	C1CW93
Deinococcus geothermalis (strain DSM 11300)	Q1J1K2	Q1IXT6
Deinococcus gobiensis (strain DSM 21396 / JCM 16679 / CGMCC 1.7299 / I-0)	H8GT97	**H8GRQ1**
Deinococcus maricopensis (strain DSM 21211 / LMG 22137 / NRRL B-23946 / LB-34)	E8U2Z4	E8U7J5
Deinococcus peraridilitoris (strain DSM 19664 / LMG 22246 / CIP 109416 / KR-200)	K9ZYH0	K9ZZF9
Deinococcus proteolyticus (strain ATCC 35074 / DSM 20540 / JCM 6276 / NBRC 101906 / NCIMB 13154 / VKM Ac-1939 / CCM 2703)	F0RQ19	F0RP80
Desmospora sp. 8437	F5SIP0	F5SJX1
Geobacillus sp. (strain Y412MC10)	D3EDH6	D3EA51
Granulicella mallensis (strain ATCC BAA-1857 / DSM 23137 / MP5ACTX8)	G8NS20	G8NRW4
Haliangium ochraceum (strain DSM 14365 / JCM 11303 / SMP-2)	D0LRJ1	D0LGP2
Koribacter versatilis (strain Ellin345)	Q1IPZ2	Q1IVA1
Marinithermus hydrothermalis (strain DSM 14884 / JCM 11576 / T1)	F2NNP7	F2NMC1
Meiothermus ruber (strain ATCC 35948 / DSM 1279 / VKM B-1258 / 21)	D3PKL1	D3PSQ6
Meiothermus silvanus	D7BI01	D7BHQ0
Mycobacterium africanum (strain GM041182)	F8M6Y9	F8M6R8
Mycobacterium bovis (strain BCG / Pasteur 1173P2)	A1KP10	A1KIC4
Mycobacterium canettii (strain CIPT 140010059)	G0TI61	G0TIE0
Mycobacterium orygis 112400015	M8DC12	M8CNW8
Mycobacterium tuberculosis XTB13-178	A0A058S963	A0A058SFT9
Myxococcus fulvus (strain ATCC BAA-855 / HW-1)	F8CD82	F8CQ87
Myxococcus stipitatus (strain DSM 14675 / JCM 12634 / Mx s8)	L7U5W9	L7UCQ5, L7U8C3
Myxococcus xanthus (strain DK 1622)	Q1D990	Q1D530, Q1DAY3
Nitrolancea hollandica Lb	I4EE09	I4ENI5, I4ECI8
Oceanithermus profundus (strain DSM 14977 / NBRC 100410 / VKM B-2274 / 506)	E4U5Y7	E4U7I0
Oscillochloris trichoides DG-6	E1IIL5	E1IFH2
Paenibacillus barengoltzii G22	R9LAX6	R9LC78

Paenibacillus curdlanolyticus YK9	E0IBW0	E0I305
Paenibacillus lactis 154	G4H881	G4H974
Paenibacillus larvae subsp. *larvae* DSM 25430	V9WF27	V9W1Z8
Paenibacillus mucilaginosus (strain KNP414)	F8FF45	F8FPG3
Paenibacillus polymyxa SQR-21	W8TVC8	-
Paenibacillus sabinae T27	X4ZAC8	X4ZUL6
Paenibacillus sp. (strain JDR-2)	C6D8P7	C6CYA3
Paenibacillus sp. oral taxon 786 str. D14	C6J7J9	C6IVA1
Paenibacillus terrae (strain HPL-003)	G7VQT0	-
Paenibacillus vortex V453	E5Z2N2	E5Z1L2
Plesiocystis pacifica SIR-1	A6FZY4	A6GIG3
Roseiflexus castenholzii (strain DSM 13941 / HLO8)	A7NN53	A7NG62
Salinisphaera shabanensis E1L3A	U2G3Z6	U2EPQ0
Solibacter usitatus (strain Ellin6076)	Q01U18	Q01P54, Q01Y58, Q02B10
Sphaerobacter thermophilus (strain DSM 20745 / S 6022)	D1C1R8	D1C1K5
Stackebrandtia nassauensis (strain DSM 44728 / NRRL B-16338 / NBRC 102104 / LLR-40K-21)	D3PUF6	D3PW85
Stigmatella aurantiaca (strain DW4/3-1)	Q093P7	E3FL00
Symbiobacterium thermophilum (strain T / IAM 14863)	Q67JI8	**Q67QA7**
Thermaerobacter marianensis (strain ATCC 700841 / DSM 12885 / JCM 10246 / 7p75a)	E6SJL1	E6SK76
Thermaerobacter subterraneus DSM 13965	K6QE59	K6NYE1
Thermobacillus composti (strain DSM 18247 / JCM 13945 / KWC4)	L0EHU3	L0ECR5
Thermomicrobium roseum (strain ATCC 27502 / DSM 5159 / P-2)	B9L0Z7	B9L1P8
Thermus aquaticus Y51MC23	B7A8F6	B7A5E9
Thermus oshimai JL-2	K7QYE5	K7QVR7
Thermus scotoductus (strain ATCC 700910 / SA-01)	E8PQM1	E8PP35
Thermus thermophilus (strain SG0.5JP17-16)	F6DDK7	F6DDI9
Tistrella mobilis (strain KA081020-065)	I3TVF4	I3TJ38
Truepera radiovictrix (strain DSM 17093 / CIP 108686 / LMG 22925 / RQ-24)	D7CSF8	D7CVM3

[a]Ubl-MoaE linear fusion protein sequences were collected from Interpro database (http://www.ebi.ac.uk/interpro/) [1], accession number and species name of each protein are listed. The JAB1/MPN+/MOV34 (JAMM) proteins were searched under family M67 (JAMM belongs to) by MEROPS-the peptidases database [2] and by UniProt database [3], the UniProt ID numbers are listed. -, not found; *not annotated but conserved based on genome coding sequence: *Sulfolobus tokodaii* GI:47118305 (1411925…1411602); *Metallosphaera cuprina* GI:329566006 (151456…151779); JAB1/MPN+/MOV34 (JAMM) proteins include RadC_JAB domain proteins. Found based on RadC-like_JAB domain (IPR025657, red), JAB domain (IPR028090, blue), and MPN super family (cl13996, green).

References for S1 Table

1. Hunter S, Jones P, Mitchell A, Apweiler R, Attwood TK, Bateman A, et al. InterPro in 2011: new developments in the family and domain prediction database. Nucleic Acids Res. 2012;40:D306-12. doi: 10.1093/nar/gkr948.

2. Rawlings ND, Waller M, Barrett AJ, Bateman A. MEROPS: the database of proteolytic enzymes, their substrates and inhibitors. Nucleic Acids Res. 2014;42:D503-9. doi: 10.1093/nar/gkt953.

3. Consortium U. Activities at the Universal Protein Resource (UniProt). Nucleic Acids Res. 2014;42:D191-8. doi: 10.1093/nar/gkt1140.
