# Supplementary material for: Ubiquitin-Like Protein SAMP1 and JAMM/MPN+ Metalloprotease HvJAMM1 Constitute a System for Reversible Regulation of Metabolic Enzyme Activity in Archaea
Source: PLoS One. 2015 May 26;10(5):e0128399. doi: 10.1371/journal.pone.0128399 (PMC4443979; doi:10.1371/journal.pone.0128399)
Supplement: S2 Table — (PDF) [file pone.0128399.s002.pdf]

S2 Table. Strains and plasmids used in this study[a].

Strain, plasmid or primer	Description	Source or reference
E. coli		
TOP10	F⁻ *recA1 endA1 hsdR17*(r_K^- m_K^+) *supE44 thi-1 gyrA relA1*	Invitrogen
GM2163	F⁻ *ara-14 leuB6 fhuA31 lacY1 tsx78 glnV44 galK2 galT22 mcrA dcm-6 hisG4 rfbD1 rpsL136 dam13*::Tn*9 xylA5 mtl-1 thi-1 mcrB1 hsdR2*	New England Biolabs
Rosetta (DE3)	F- *ompT hsdS*$_B$(r_B^- m_B^-) *gal dcm* (DE3) pRARE (Cam^R)	Novagen
Hfx. volcanii		
DS70	wild-type isolate DS2 cured of plasmid pHV2	[1]
H26	DS70 *ΔpyrE2*	[2]
HM1052	H26 *ΔubaA*	[3]
SC116	H26 *ΔubaA Δjamm1*	This study
SC100	H26 *Δjamm1*	This study
HM1109	H26 *Δsamp1ΔmoaE*	[4]
NH01	H26 *Δsamp1ΔmoaEΔjamm1*	This study
**Plasmids**		
pTA131	Ap^r; pBluescript II carries P_{fdx}-*pyrE2* with MCS	[2]
pET24b	Kan^r; expression vector	Novagen
pJAM202c	Ap^r; Nv^r; *Hfx. volcanii-E. coli* shuttle plasmid, empty vector control	[5]
pJAM809	Amp^r Nv^r; pJAM202 containing $P2_{rrnA}$-*hvo1862-strepII* (*KpnI* site upstream of StrepII coding sequence)	[6]
pJAM1314	Ap^r; Nv^r; Flag-SAMP1 and MoaE-StrepII	[4]
pJAM1796	Ap^r; Nv^r; Linear Flag-SAMP1-MoaE M1A-StrepII	[4]
pJAM1804	Ap^r; Nv^r; Linear Flag-SAMP1ΔGG-MoaE M1A-StrepII	[4]
pJAM1807	Ap^r; Nv^r; Linear Flag-SAMP1ΔVSGG-MoaE-StrepII	[4]
pJAM2707	Ap^r; Nv^r; Flag-SAMP1 and MoaE K240R-StrepII	This study
pJAM2708	Ap^r; Nv^r; Flag-SAMP1 and MoaE K247R-StrepII	This study
pJAM2709	Ap^r; Nv^r; Flag-SAMP1 and MoaE K248R-StrepII	This study
pJAM1767	Ap^r; pTA131-based pre-knockout plasmid for *hvo_2505* (HvJAMM1)	This study
pJAM2300	Ap^r; pTA131-based knockout plasmid for *hvo_2505* (HvJAMM1)	This study
pJAM991	Ap^r; pET15b-derived, His-HvJAMM1	[4]
pJAM2302	Kan^r; pET24b-derived, Flag-SAMP1-MoaE-StrepII linear fusion	This study
pJAM2303	Kan^r; pET24b-derived, MoaE-StrepII	This study

[a]Abbreviations: Ap^r, ampicillin resistance; Kan^r, kanamycin resistance; Nv^r, novobiocin resistance; Cam^R, chloramphenicol resistance; *-strepII*, C-terminal StrepII-tag fusion coding sequence (preceded by a KpnI site that facilitated generation of the C-terminal StrepII fusion with GlyThr linker). *E. coli* TOP10 was used for routine recombinant DNA analysis. *E. coli* GM2163 was used for preparation of plasmid DNA prior to transformation of *Hfx. volcanii* by standard methods [7]. *E. coli* Rosetta (DE3) was used for heterologous expression of His-HvJAMM1.

References for S2 Table

1. Wendoloski D, Ferrer C, Dyall-Smith ML. A new simvastatin (mevinolin)-resistance marker from *Haloarcula hispanica* and a new *Haloferax volcanii* strain cured of plasmid pHV2. Microbiology. 2001;147(Pt 4):959-64.

2. Allers T, Ngo HP, Mevarech M, Lloyd RG. Development of additional selectable markers for the halophilic archaeon *Haloferax volcanii* based on the *leuB* and *trpA* genes. Appl Environ Microbiol. 2004;70(2):943-53.

3. Miranda H, Nembhard N, Su D, Hepowit N, Krause D, Pritz J, et al. E1- and ubiquitin-like proteins provide a direct link between protein conjugation and sulfur transfer in archaea. Proc Natl Acad Sci U S A. 2011;108(11):4417-22. doi: 1018151108 [pii]10.1073/pnas.1018151108.

4. Hepowit NL, Uthandi S, Miranda HV, Toniutti M, Prunetti L, Olivarez O, et al. Archaeal JAB1/MPN/MOV34 metalloenzyme (HvJAMM1) cleaves ubiquitin-like small archaeal modifier proteins (SAMPs) from protein-conjugates. Mol Microbiol. 2012;86(4):971-87. doi: 10.1111/mmi.12038.

5. Zhou G, Kowalczyk D, Humbard M, Rohatgi S, Maupin-Furlow J. Proteasomal components required for cell growth and stress responses in the haloarchaeon H*aloferax volcanii.* J Bacteriol. 2008;190(24):8096-105.

6. Humbard MA, Zhou G, Maupin-Furlow JA. The N-terminal penultimate residue of 20S proteasome α1 influences its Nα acetylation and protein levels as well as growth rate and stress responses of H*aloferax volcanii.* J Bacteriol. 2009;191(12):3794-803. doi: 10.1128/JB.00090-09.

7. Dyall-Smith M. The Halohandbook: Protocols for Halobacterial Genetics. Dyall-Smith M, editor. http://www.haloarchaea.com/resources/halohandbook/Halohandbook_2009_v7.2mds.pdf2009.
