# Supplementary material for: Ubiquitin-Like Protein SAMP1 and JAMM/MPN+ Metalloprotease HvJAMM1 Constitute a System for Reversible Regulation of Metabolic Enzyme Activity in Archaea
Source: PLoS One. 2015 May 26;10(5):e0128399. doi: 10.1371/journal.pone.0128399 (PMC4443979; doi:10.1371/journal.pone.0128399)
Supplement: S3 Table — (PDF) [file pone.0128399.s003.pdf]

S3 Table. Primers used in this study[a].

Primer Pair	Primer Sequence	PCR product/description	Source or reference
NdeI-Flag-KpnI fwd<br>StrepII-KpnI rev	5'- tggcctcatatgGACTACAAGGACGACGACGAC AAGggtacc-3'<br>5'- CTGCGGGTGGCTCCAggtacc-3'	Flag-SAMP1-MoaE-StrepII linear fusion	This study
HVO_1864 K240R inverse fwd<br>HVO_1864 K240R inverse rev	5'-CATCGACCGCCTGcggGACGAGGTGCCG-3'<br>5'-CCGTCTTCGACCGTCCGGAACGC-3'	MoaE K240R-StrepII	This study
HVO_1864 K247R inverse fwd<br>HVO_1864 K247/248 gen inv rev	5'-tttcggAAGGAGACGACCACCGACG-3'<br>5'-AATCGGCACCTCGTCTTTCAGGCGG-3'	MoaE K247R-StrepII	This study
HVO_1864 K248R inverse fwd<br>HVO_1864 K247/248 gen inv rev	5'-tttaagcggGAGACGACCACCGACG-3'<br>5'-AATCGGCACCTCGTCTTTCAGGCGG-3'	MoaE K248R-StrepII	This study
HVO_1864 K247,248R inv fwd<br>HVO_1864 K247/248 gen inv rev	5'-tttcgccggGAGACGACCACCGACG-3'<br>5'-AATCGGCACCTCGTCTTTCAGGCGG-3'	MoaE K247,248R-StrepII	This study
HVO_2505 NdeI up fwd<br>HVO_2505 stop BlpI dwn rev	5'-ttccgggcatatgACTTCGAGTAGGCTCTCTCTC-3'<br>5'-aatgctcagcTCACTCGACGGCGACGGAGAG-3'	HvJAMM1	[1]
Hvo_2505 511 BamHI fwd<br>Hvo_2505 521 HindIII rev	5'-atggatccGCAGCGCAGGACGTCAT-3'<br>5'-gcgaagcttGAGACGTTGACGACGC-3'	*hvjamm1* and 0.5 kb flanking 5' and 3' of this gene; includes BamHI and HindIII sites for cloning into pTA131 to generate pJAM1767	[1]
HVO_2505-inverse down fwd<br>HVO_2505-inverse up rev-new	5'-TCGGTCCTCGGCCAGTCGCC-3'<br>5'- CTACTCGAAGTCACGGCGCATGGCG-3'	*hvjamm1*-knockout plasmid pJAM2300 generated by inverse PCR; pJAM1767 as template	This study

[a]Lowercase letters represent DNA sequence introduced to facilitate ligation of PCR product and/or site-directed mutagenesis.

References for S3 Table

1. Hepowit NL, Uthandi S, Miranda HV, Toniutti M, Prunetti L, Olivarez O, et al. Archaeal JAB1/MPN/MOV34 metalloenzyme (HvJAMM1) cleaves ubiquitin-like small archaeal modifier proteins (SAMPs) from protein-conjugates. Mol Microbiol. 2012;86(4):971-87. doi: 10.1111/mmi.12038.
